# Fabrication and Characterization of Bio-Based Aerogels Derived from *Bacillus amyloliquefaciens* SQ-2 Exopolysaccharides: Structural Characterization and In Vitro Antitumor Activity Analysis

**DOI:** 10.3390/gels12060462

**Published:** 2026-05-25

**Authors:** Tianjiao Zhao, Lei Huang, Sihan Wei, Chengci Liu, Jinhua Xu, Lu Qiao, Jincheng Li, Chaoying Zhang, Yingchun Mu, Zhiyang Zhao, Meitong Li, Xin Hu

**Affiliations:** 1 Tianjin Key Laboratory of Organic Solar Cells and Photochemical Conversion, College of Chemistry and Chemical Engineering, Tianjin University of Technology, Tianjin 300384, China; tianjiaozhao@stud.tjut.edu.cn (T.Z.); huanglei@tjut.edu.cn (L.H.); yj20251014@stud.tjut.edu.cn (S.W.); chengciliu@163.com (C.L.); 2 Chinese Academy of Fishery Sciences, Beijing 100141, China; xujh@cafs.ac.cn (J.X.); qiaolu@cafs.ac.cn (L.Q.); lijc@cafs.ac.cn (J.L.); cyzhang0317@cafs.ac.cn (C.Z.); muyc@cafs.ac.cn (Y.M.); 3 Hainan Fisheries Innovation Research Institute, Chinese Academy of Fishery Sciences, Sanya 572000, China; 4 Jiangsu Key Laboratory of New Energy Devices & Interface Science, School of Chemistry and Materials Science, Nanjing University of Information Science and Technology, Nanjing 210044, China; zhzh@nuist.edu.cn

**Keywords:** aerogel, exopolysaccharide, *Bacillus amyloliquefaciens*, structural characterization, antitumor activity

## Abstract

Aerogels derived from microbial exopolysaccharides are useful in the food, pharmaceutical, and environmental sectors, but their application in anticancer therapy is constrained by inadequate characterization, especially regarding effects on normal cells. This study used ethanol precipitation and trichloroacetic acid deproteinization to extract crude exopolysaccharide from the fermentation broth of *Bacillus amyloliquefaciens* SQ-2. The pure fraction, EPS-3791, was obtained using Sephadex G-100 gel filtration chromatography and DEAE cellulose ion exchange. The weight–average molecular weight of EPS-3791 was 64.4 kDa. Monosaccharide analysis indicated fructan as the dominant component, which was consistent with the results of methylation analysis and NMR spectroscopy, confirming that EPS-3791 is a fructan mainly linked by →1)–Fruf–(2→bonds. UV scanning indicated high purity. FTIR analysis revealed functional groups including hydroxyl, carbonyl, and C–O–C groups. EPS-3791 exhibited a porous three-dimensional network morphology by SEM, with a decomposition temperature of 191.61 °C by TGA. Additionally, aerogels were prepared by freeze drying. EPS-3791 aerogels demonstrated minimal cytotoxicity to normal L929 cells while inhibiting the growth of human lung cancer A549, breast cancer MCF–7, and cervical cancer HeLa cells in a dose-dependent manner. Scratch wound healing experiments revealed that EPS-3791 aerogels hindered HeLa cell migration while promoting L929 wound closure. These findings identify EPS-3791 as a fructan-type exopolysaccharide aerogel with specific anticancer properties.

## 1. Introduction

Microbial exopolysaccharides (EPSs) are utilized in the food, environmental, and pharmaceutical industries [1,2,3] because of their biocompatibility and diverse bioactivities, including antioxidative, antibacterial, and anticancer properties [4]. The structural characteristics of EPSs determine their functional properties [5], and microbial manufacturing provides advantages such as cheaper fermentation costs and simpler purifying techniques relative to plant- or animal-derived polysaccharides [6]. Nonetheless, the EPSs from many newly isolated microbial strains have not been structurally characterized, and their biological activities remain unexplored.

The U.S. Food and Drug Administration (FDA) has deemed *Bacillus amyloliquefaciens*, a Gram-positive bacteria, safe for use in food fermentation and agricultural biocontrol [7]. Several studies have reported biological activities of the EPS produced by *B. amyloliquefaciens* strains. Chen et al. found that *B. amyloliquefaciens* EPS modulated glycemic levels in mice and promoted cellular glucose uptake via Akt activation [8] Zhao et al. isolated an antioxidant EPS from *B. amyloliquefaciens* GSBa-1 with radical scavenging capacity [9], and Yang et al. reviewed the structural characteristics and biotechnological applications of EPS from *Bacillus* species [7]. Strain SQ-2, identified as *B. amyloliquefaciens*, was recently shown to exhibit biocontrol activity against *Aspergillus tubingensis* on table grapes through the production of antimicrobial metabolites [9,10], but the EPS produced by this strain has not been characterized or evaluated for bioactivity.

The antitumor potential of EPS from *Bacillus* and related species has been documented in several studies. Chen et al. reported that the EPS from an endophytic *B. amyloliquefaciens* isolate exhibited concentration-dependent cytotoxicity against gastric carcinoma MC–4 and SGC–7901 cells, with IC50 values of 19.7 and 26.8 µg/µL, respectively, through mitochondrial dysfunction [11]. El-Newary et al. found that acidic EPS from a marine *B. amyloliquefaciens* 3MS 2017 strain inhibited MCF–7 breast cancer and PC3 prostate cancer cell proliferation [12]. Additionally, it has been noted that bacterial EPS is selective for cancer cells. According to Farag et al., *Bacillus mycoides* EPS reduced the cytotoxicity of normal BHK cells while inhibiting the proliferation of HepG2 and Caco-2 cells [13]. Wahab et al. found that co-cultured bacterial levan inhibited the development of HepG2 cells by 70.70% and reduced cancer cell migration [14]. However, the structural basis for anticancer action of *B. amyloliquefaciens*-derived EPS, particularly fructan-type polysaccharides, has yet to be determined. Beyond solution-phase bioactivity studies, there is growing interest in converting microbial polysaccharides into aerogel-form biomaterials. Freeze drying aqueous EPS solutions generate porous three-dimensional scaffolds that retain the chemical functionality of the native polymer while acquiring the high surface area and low-density characteristic of aerogels [15]. The high surface area and porous architecture of such scaffolds can increase contact between bioactive polysaccharide chains and target cells, opening a route toward biomaterial applications of microbial EPS.

This study reports the isolation, structural characterization, aerogel fabrication, and in vitro anticancer evaluation of EPS from *Bacillus amyloliquefaciens* SQ-2. Crude EPS was recovered from fermentation broth by ethanol precipitation and TCA deproteinization, subsequently refined using Sephadex G-100 gel filtration chromatography and DEAE–cellulose ion exchange. The purified fraction EPS-3791 was characterized by FTIR, methylation analysis, NMR, SEM, AFM, XPS, and TGA. Freeze drying was used to create EPS-3791 aerogels, whose antiproliferative and antimigratory properties were assessed in vitro against human lung cancer A549, breast cancer MCF–7, and cervical cancer HeLa cells. As a selectivity control, normal L929 fibroblasts were employed. Together, these results establish EPS-3791 as a fructan with selective anticancer properties and demonstrate its feasibility as an aerogel scaffold.

## 2. Results and Discussion

### 2.1. Extraction and Purification of EPS-3791

**Figure 1:**
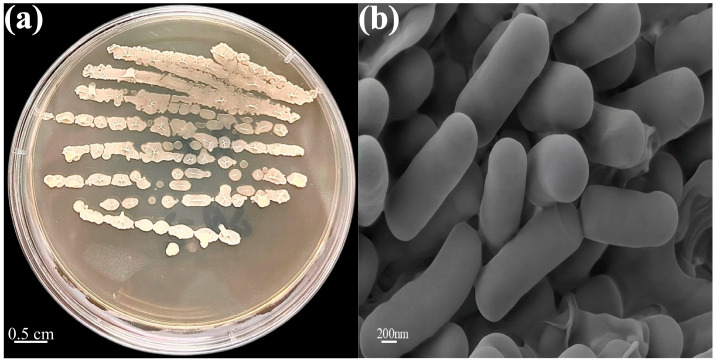
Flat colony morphology of strain SQ-2 on LB solid medium (**a**); SEM image of SQ-2 (**b**).

**Figure 2:**
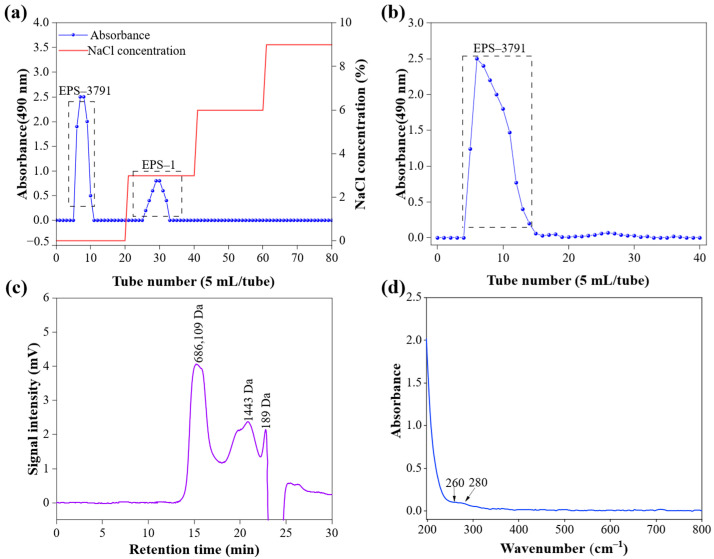
Elution curves of EPS on DEAE cellulose column (**a**) and Sephadex G-100 glucan gel column (**b**). HPGPC of EPS-3791 (**c**) and UV–VIS spectrum of EPS-3791 (**d**).

Strain SQ-2 formed smooth, circular colonies on LB solid medium (Figure 1a), and SEM observation revealed rod-shaped cells with intact cell surfaces (Figure 1b). Crude exopolysaccharides were isolated from strain SQ-2 by ethanol precipitation, TCA deproteinization, dialysis and lyophilization. According to Equations (1) and (2), the yield of EPS and the preparation yield of aerogel were 566.5 mg/L and 93.6%, respectively. The crude exopolysaccharides were first fractionated by DEAE–cellulose ion exchange chromatography, with stepwise elution using ultrapure water and 3%, 6%, 9% NaCl solutions (Figure 2a). The neutral fraction EPS-3791 was collected and further purified by Sephadex G-100 gel filtration chromatography (Figure 2b). For further analysis, the purified EPS-3791 was lyophilized and dialyzed. The results of high-performance gel permeation chromatography (HPGPC) analysis (Figure 2c) showed that the chromatogram of EPS-3791 presented three distinct elution peaks with retention times of approximately 15 min, 20 min, and 22 min, respectively. Calibrated against dextran standards, the apparent weight–average molecular weight of the main component was estimated to be 598,254 Da, while the other two minor peaks corresponded to small-molecular-weight fragments of 5864 Da and 230 Da, respectively. The absolute molecular weight determined subsequently by SEC–MALS–RI was 64.4 kDa; the discrepancy reflects the overestimation inherent in standard-calibrated HPGPC when the hydrodynamic behavior of the analyte differs from that of the calibrants. The average molecular weight measured (Mw) was 64.4 kDa with a root mean square (RMS) radius of 18.0 nm and a polydispersity index (PDI) (Mw/Mn) of 1.6. EPS-3791 displayed a relatively broad molecular weight distribution. A PDI value closer to 1 indicated a narrower distribution, so the values > 1 reflected increasing polydispersity [16,17].

In the UV–VIS spectrum (UV100–star, INESA Scientific Instrument Co., Ltd., Shanghai, China), the purified EPS-3791 displayed a single peak and did not exhibit any noticeable absorption peaks at 260 or 280 nm, indicating the absence of nucleic acids and proteins (Figure 2d). The total sugar content in the purified EPS-3791 was 86.22% (*w*/*w*); this value exceeds that reported for many microbial EPS [18]. Subsequently, EPS-3791 was dialyzed and freeze dried prior to structural and functional characterization.

### 2.2. Structure of EPS-3791

#### 2.2.1. Monosaccharide Composition Analysis

**Figure 3:**
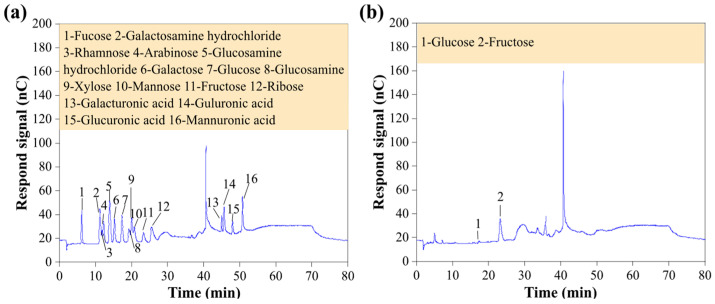
IC analysis of standard monosaccharides (**a**) and EPS-3791 (**b**).

High-performance anion exchange chromatography with pulsed amperometric detection (HPAEC–PAD) was used to assess the monosaccharide composition of EPS-3791 (Figure 3a,b). Detailed peak area, retention time, and molar ratio data are provided in Table S1. Fructose was the primary component (95.28%), with minor amounts of glucose (4.72%). Therefore, EPS-3791 is in fact a fructan.

#### 2.2.2. FTIR Spectral Analysis

FTIR spectroscopy was used to identify functional groups in EPS-3791 (Figure S1). The spectrum was compared with the FTIR spectra of typical microbial fructans. The broad, powerful band at 3409 cm^−1^ was provisionally assigned to O–H stretching vibrations, which are compatible with numerous hydroxyl groups and hydrogen bonding. The band at 2932 cm^−1^ corresponded to aliphatic C–H stretching vibrations. The absorption near 1644 cm^−1^ was attributed to the stretching vibration of C=O groups. The signals at 1533 and 1451 cm^−1^ were assigned to C–O stretching vibrations [19]. A weak band at 1250 cm^−1^ was attributable to C–O–C stretching or C–H deformation modes, consistent with observations in other microbial polysaccharides [20]. The fingerprint region between 1200 and 1000 cm^−1^ was dominated by C–O–C and C–O–H vibrations, which are suggestive of pyranose ring structures. This was highly consistent with the characteristic fingerprint region of typical fructans [20,21,22,23,24,25]. The asymmetrical stretching vibration of the pyranose ring was associated with the band at 947 cm^−1^. The band at 882 cm^−1^ suggested the presence of a β-glycosidic link between the sugar residues [23]. The overall FTIR spectral pattern of EPS-3791 was consistent with those of known fructans, further confirming that EPS-3791 is a fructan polysaccharide [19,21]. A band about 819 cm^−1^ indicated that the mannose unit had an α-type structure [24]. Additionally, the absence of an absorption peak at 1700–1750 cm^−1^ indicated that uronic acids were not present in EPS-3791 [23], consistent with the monosaccharide composition studied.

#### 2.2.3. Methylation Analysis of EPS-3791

To better understand the polymer structure of EPS-3791, methylation was performed using gas chromatography–mass spectrometry (GC–MS) analysis [26]. The corresponding EI mass spectra of each partially methylated alditol acetate (PMAA) derivative and complete methylation analysis data are presented in Figure S2, Figure S3, Table S2, and Table S3, respectively. Table S2 and Figure S2 show detailed information about retention time, methylated sugar fragments, mass fragments, molar ratios, and glycosidic bond types. A total of five distinct methylated derivatives were detected in EPS-3791, including 1,3,4,5–Me_4_–Manf/Glcf, 2,3,4,6–Me_4_–Glcp, 3,4,5–Me_3_–Manf/Glcf, 2,3,4–Me_3_–Glcp, and 2,4–Me_2_–Glcp. These data indicated that the native polysaccharide was composed predominantly of fructose and glucose residues. During methylation hydrolysis reduction procedures, as one of the typical ketose residues, fructose is isomerized into glucose and mannose [27,28]. Methylated alditol derivatives derived from fructose may appear as glucopyranosides and furanose mannopyranoside. Consequently, the inferred linkage types included Fruf–(2→, Glcp–(1→, →1)–Fruf–(2→, →6)–Glcp–(1→, and →3,6)–Glcp–(1→, with molar ratios of 0.095: 0.015: 0.777: 0.014: 0.099. Therefore, EPS-3791 can be classified as a fructan, with the predominant linkage assigned as →1)–Fruf–(2→.

Cai et al. characterized a levan-type EPS from *B. amyloliquefaciens* JN4 (EPS-JN4) whose main chain consisted of beta–(2,6)-linked Fruf residues with single–residue branches at every six residues [29]; the →1)–Fruf–(2→ backbone of EPS-3791 represents a different fructan variant within the same species. Yang et al. reported two EPS fractions from *B. amyloliquefaciens* C–1 with molecular weights of 79.6 kDa (EPS-1, glucose/mannose/galactose/arabinose = 15:4:2:1) and 19.8 kDa (EPS-2, glucose/mannose = 3:1) [30]; neither was identified as a fructan, indicating that fructan production by *B. amyloliquefaciens* is likely strain-dependent and influenced by culture conditions.

#### 2.2.4. NMR Analysis

**Figure 4:**
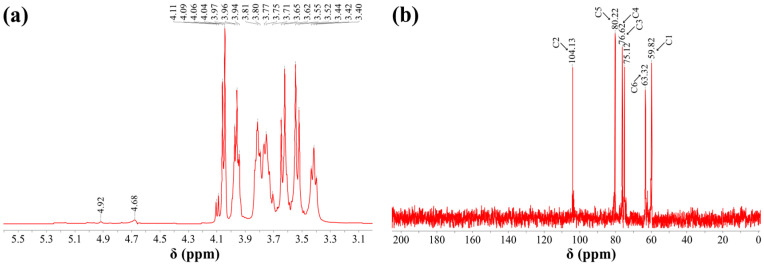
NMR of EPS-3791. (**a**) ^1^H NMR; (**b**) ^13^C NMR.

One-dimensional nuclear magnetic resonance (NMR) spectroscopy was used to better clarify the structural properties of EPS-3791 (Figure 4). The ^1^H NMR spectrum of EPS-3791 predominantly exhibited resonances between δ 3.0 and 5.5 ppm, a range characteristic of carbohydrate proton signals in polysaccharides (Figure 4a). A series of ^13^C NMR signals between δ 60 and 80 ppm were assigned to ring carbons C2 to C6 of the sugar residues: specifically, C2 at δ 59.82 ppm, C3 at δ 60.32 ppm, C4 at δ 62.10 ppm, C5 at δ 75.12 ppm, and C6 at δ 63.32 ppm (consistent with the chemical shift characteristics of fructose ring carbons) (Figure 4b).

Combined monosaccharide and methylation data indicated that EPS-3791 was composed mainly of fructose with minor glucose. Fructose accounted for 95.28% of monosaccharides detected. According to relevant literature, the C1 chemical shift of fructopyranose or pyranofructose derivatives is 64.7 ppm [31]. A signal at δ 64.7 ppm was observed in the ^13^C NMR spectrum of EPS-3791, confirming the presence of fructopyranose moieties in the polysaccharide backbone. Similarly, the chemical shifts for the remaining ring carbons (C2 to C6) agree well with previously reported values for fructose residues, where C2–C6 of fructopyranose typically resonate within the δ 58–78 ppm range. Taken together, these 1D NMR data were fully consistent with the methylation and monosaccharide analyses, supporting the assignment of EPS-3791 as a fructan with the predominant linkage mode β–D–Fruf–(2→ (Figure S4).

#### 2.2.5. Microstructural Analysis

**Figure 5:**
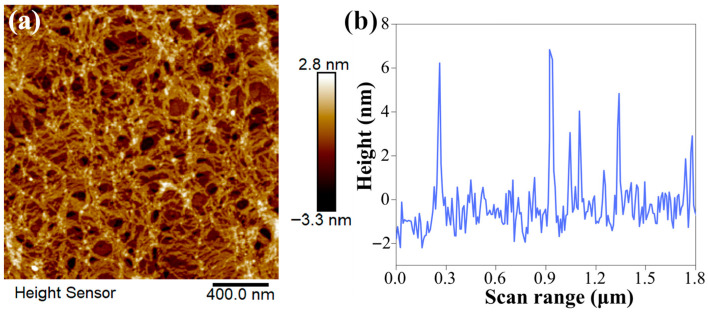
AFM images of EPS-3791. Two-dimensional (2D) image (**a**) and height distribution (**b**) for 10 μg/mL of EPS-3791 solution.

The planar AFM image (Figure 5a) showed globular aggregates with an average particle size of 400 nm, while the three-dimensional stereoscopic image showed mountain-like lumps with nearly uniform size, similar to the EPS produced by *Lactobacillus sanfranciscensis* Ls-1001 [32]. This suggests that the EPS-3791 may have similar homogeneity to Ls-1001. Furthermore, the height of EPS-3791 aggregates varied from −3.3 nm to 2.8 nm. The height distribution curve (Figure 5b) further indicated that the vertical dimension of the aggregates was mainly distributed in the range of 0–6 nm within the 0–1.6 μm scan range. Based on statistically analyzed particles (Figure S5), the average size of EPS-3791 aggregates were (44.65 ± 12.97) nm.

**Figure 6:**
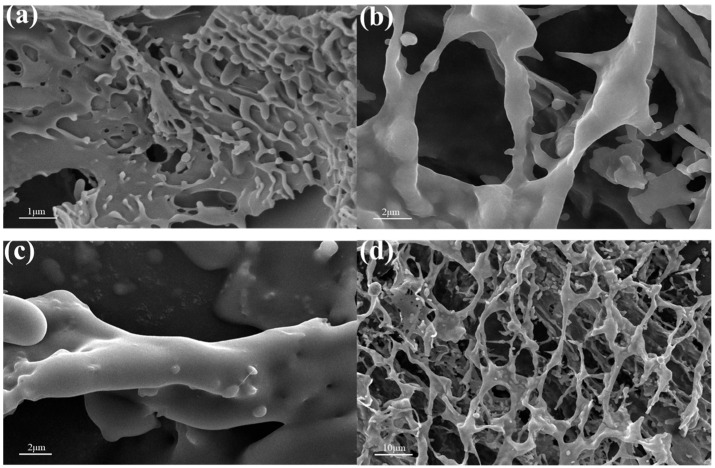
SEM images of EPS-3791 at ×10,000 (**a**), ×5000 (**b**,**c**), and ×1000 (**d**).

The micromorphology and surface characteristics of EPS-3791 were investigated using scanning electron microscopy (SEM). As depicted in Figure 6a–d, EPS-3791 exhibited a uniform, porous three-dimensional network with interconnected fibrous and globular substructures; the pore diameters ranged from 1 to 10 um. According to the quantitative statistical results in Figure S6, the average pore size of EPS-3791 was calculated to be 9.76 μm. Marimuthu et al. reported that the EPS produced by *Bacillus subtilis* EPS003 exhibited a dense matrix with porous properties, and its water-holding capacity was 285.7 ± 3.8% [33]. As a fructan produced by *Bacillus amyloliquefaciens* SQ-2, EPS-3791 is rich in hydroxyl groups, which can form strong hydrogen bonds with water molecules and exhibit obvious hydration and swelling behavior in aqueous solution [34]. Furthermore, the porous network structure observed in SEM images provides abundant internal voids for physical adsorption and retention of water. Fructans generally possess excellent water retention and moisture retention properties [35,36]. Therefore, EPS-3791 also possesses favorable water-holding capacity.

#### 2.2.6. XPS Analysis of EPS-3791

**Figure 7:**
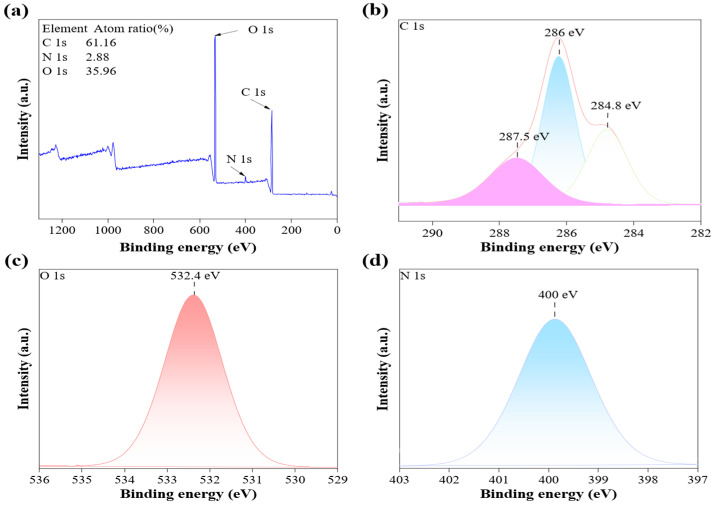
The wide spectra (**a**) and high-resolution XPS peaks of C1s (**b**), O1s (**c**), and N1s (**d**).

EPS-3791’s surface elemental composition and chemical bonding states were examined using X-ray photoelectron spectroscopy (XPS). The atomic percentages of C, N, and O were 61.16%, 2.88%, and 35.96%, respectively (Figure 7a). Three deconvoluted peaks, belonging to C=O, C–O–C, and C–H/C–C groups, were observed at binding energies of 287.5, 286.0, and 284.8 eV in the high-resolution C1s spectra (Figure 7b). The O1s spectrum exhibited a band near 532.4 eV that could be deconvoluted into C–O and C=O contributions (Figure 7c).The N1s region displayed a single peak at 400.0 eV (Figure 7d), attributable to amide or amine nitrogen. XPS analysis showed that EPS-3791 contained C (61.16%), O (35.96%), and trace N (2.88%), an elemental composition close to that of the EPS from *Bacillus enclensis* AP–4 [37]. Nitrogen is likely derived from residual protein in the polysaccharide matrix, consistent with the N1s binding energy at 400.0 eV assigned to amide or amine nitrogen [38]. FTIR and XPS data jointly confirmed hydroxyl and carbonyl/carboxyl groups; the trace nitrogen detected by XPS is consistent with residual protein in the polysaccharide matrix. The total sugar content of the purified EPS-3791 was 86.22% (*w*/*w*), indicating relatively high purity. According to previous studies on microbial EPSs [39,40], EPSs with high total sugar content and low impurity levels are generally considered suitable for in vitro cell experiments. Such trace impurities would not significantly interfere with the results of cell proliferation and scratch wound healing assays. Therefore, the purity of EPS-3791 is sufficient to ensure the reliability of subsequent tests.

#### 2.2.7. TG Analysis of EPS-3791

Thermogravimetric analysis was used to evaluate EPS-3791’s thermal stability (Figure S7). Three different phases of weight reduction were shown by the TG curve. The evaporation of remaining moisture was thought to be the cause of the initial weight loss below 100 °C. The major decomposition occurred in the second stage, with the DTG curve showing a maximum degradation rate at 191.61 °C, corresponding to the thermal decomposition of polysaccharide chains through depolymerization and dehydration reactions. Above 300 °C, continued gradual weight loss was observed, reflecting the carbonization of the residual material. These results indicated that EPS-3791 possessed moderate thermal stability [41].

### 2.3. Antiproliferative Activity of EPS-3791 Aerogel

**Figure 8:**
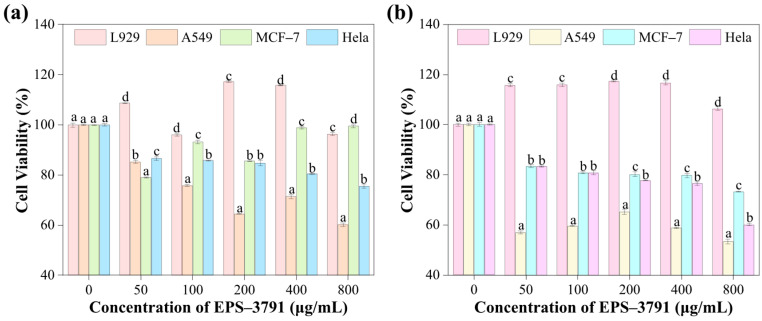
Cell viability (%): (**a**) shows the cell viability results after 24 h of EPS-3791 aerogel treatment, and (**b**) shows the results after 48 h of treatment. The letters are sorted from low to high as a < b < c < d. Significant changes between cell lines at the same concentration are indicated by different letters (Duncan’s multiple range test, *p* < 0.05), whereas no significant difference is shown by the same letters (*p* > 0.05). The data presented as mean ± SD of replicates of three independent replicates (*n* = 3). L929 is normal mouse fibroblast cells, A549 is human lung adenocarcinoma cells, MCF–7 is human breast cancer cells, and HeLa is human cervical cancer cells.

Cell viability at 24 h and 48 h is shown in Figure 8a,b. EPS-3791 aerogel demonstrated minimal cytotoxicity against normal L929 fibroblasts while inhibiting the proliferation of A549, MCF–7, and HeLa cancer cells in a dose-dependent manner. At 48 h, cell viability dropped to 53.3%, 73.3%, and 60.1% for A549, MCF–7, and HeLa at 800 μg/mL, respectively, whereas L929 viability showed no reduction, remaining ≥96% at 24 h and ≥106% at 48 h across all concentrations tested. Notably, the IC_50_ values of EPS-3791 against these cancer cell lines were all higher than 800 µg/mL. This concentration is suitable for in vitro antitumor activity comparison and owns credible biological significance. Although it exhibited relatively weak direct cytotoxicity compared with other reported bacterial EPSs, remarkable suppression on cancer cell proliferation and migration was still detected, which is typical for natural microbial polysaccharides [42,43]. A549 cells were the most sensitive of the three cancer lines; the 46.7% inhibition rate observed at 48 h/800 μg/mL is notable given the concentration range used. The aerogel form of EPS-3791 may facilitate sustained interaction between the polysaccharide and cancer cell surfaces relative to compact powder preparations, contributing to the observed activity. A comparable selectivity pattern has been reported for other bacterial EPS: Farag et al. found that EPS from *Bacillus mycoides* suppressed HepG2 and Caco-2 proliferation (IC50 = 138 and 159 μg/mL) with substantially lower toxicity toward normal BHK cells (IC50 = 254 μg/mL) [13]. Chen et al. documented concentration-dependent cytotoxicity of *B. amyloliquefaciens* EPS against gastric carcinoma MC–4 and SGC–7901 cells (IC50 = 19.7 and 26.8 μg/μL) [11], and El-Newary et al. reported inhibition of MCF–7 proliferation by acidic EPS from a marine *B. amyloliquefaciens* strain [12]. Cao et al. showed that the marine bacterial polysaccharide EPS11 blocked A549 cell adhesion through elevated Bax/Bcl-2 ratio and caspase activation [44], while Zhang et al. found that selenium-enriched polysaccharides from *Pleurotus ostreatus* inhibited multiple cancer lines via concomitant modulation of Bax/Bcl-2 signaling [45]. Whether EPS-3791 aerogel engages analogous apoptotic pathways can be tested by measuring Bax/Bcl-2 ratios and caspase-3 activation in the three cancer lines.

### 2.4. Wound Healing Assay 

**Figure 9:**
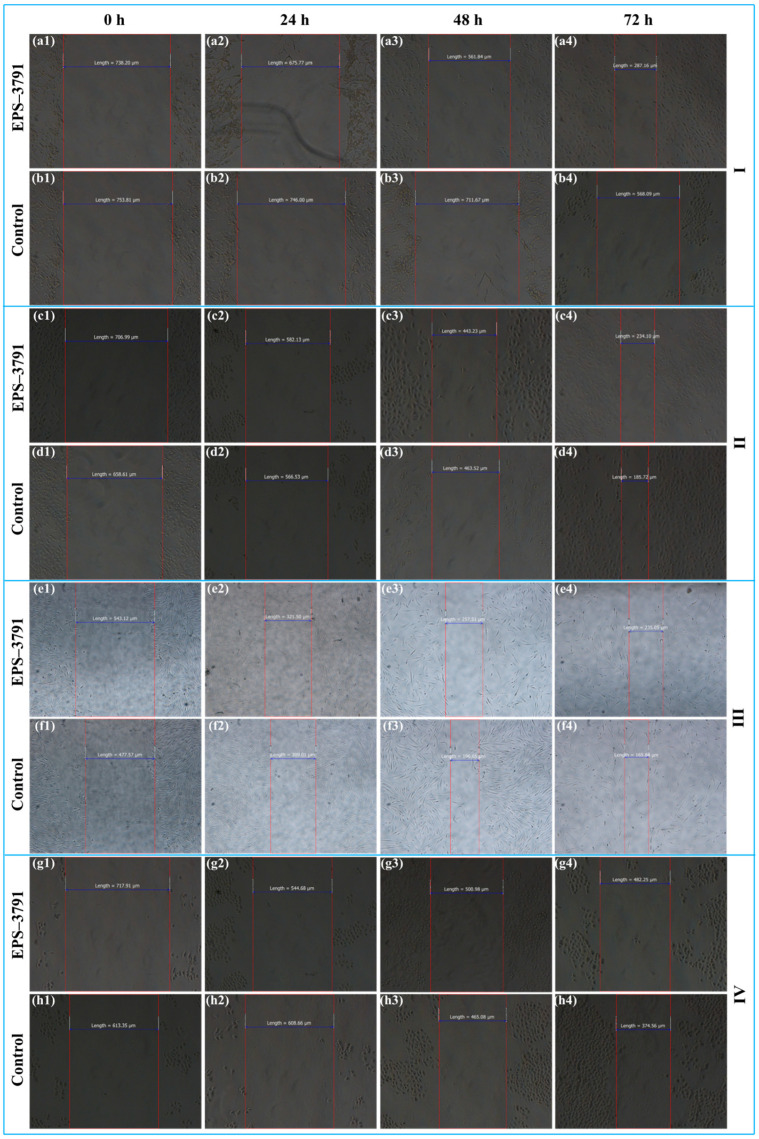
Scratch wound healing assay under an inverted microscope at various incubation times. L929 cells treated with 10 mg/mL EPS-3791 aerogel (**a1**–**a4**) and PBS medium (**b1**–**b4**); A549 cells treated with 10 mg /mL EPS-3791 aerogel (**c1**–**c4**) and PBS medium (**d1**–**d4**); MCF–7 cells treated with 10 mg/mL EPS-3791 aerogel (**e1**–**e4**) and PBS medium l (**f1**–**f4**); HeLa cells treated with 10 mg/mL EPS-3791 aerogel (**g1**–**g4**) and PBS medium l (**h1**–**h4**).

**Figure 10:**
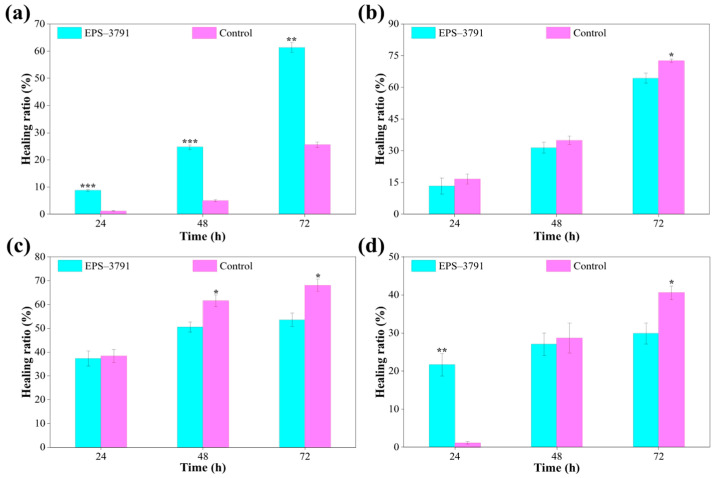
Healing ratios (%) of L929 (**a**), A549 (**b**), MCF–7 (**c**), and HeLa cells (**d**) at different incubation times. The data presented as mean ± SD of replicates. Significant differences were assessed by *t*-test (* *p* < 0.05, ** *p* < 0.01, *** *p* < 0.001).

The adherent L929, A549, MCF–7, and Hela cells were treated with 10 mg of EPS-3791 aerogel. As the IC_50_ of EPS-3791 is above 800 μg/mL, this concentration exhibited no obvious cytotoxicity and ensured reliable migration detection. As a high-molecular-weight EPS with weak cytotoxicity, relatively high concentrations are often required to observe significant migration inhibition [46,47]. The area of wound healing was monitored by capturing images every 24 h for 3 consecutive days, and wound closure was recorded using microscopic images at 100× magnification (Figure 9). For L929 cells, both the EPS-3791 aerogel-treated group and the control group exhibited gradual wound closure over time (Figure 9(a3,b3)). As clearly shown in Figure 10a, the wound healing rate of the EPS-3791 aerogel-treated group was significantly higher than that of the control group (*p* < 0.001), reaching 24.7%. At 72 h, the healing rate of the treated group further increased to 61.4%. These findings show that the EPS-3791 aerogel considerably accelerates cell wound healing rather than inhibiting the migration and proliferation of normal human cells. Furthermore, the control groups of the remaining three cell lines exhibited gradual wound closure with prolonged incubation time. In contrast, EPS-3791 aerogel treatment substantially reduced wound healing in A549, MCF–7, and HeLa cells. At the conclusion of the observation period, the healing rates for A549, MCF–7, and HeLa cells in the EPS-3791 aerogel-treated groups were 64.27%, 53.54%, and 29.91%, respectively (Figure 10b–d). These rates were considerably lower than those of the control groups (*p* < 0.05). Notably, during the early stages of incubation, the therapy significantly inhibited the HeLa cells’ ability to heal (*p* < 0.01). Xu et al. reported that endophyte-derived EPS inhibited MC–4 gastric cancer cell migration in a dose-dependent manner through downregulation of MMP2 and MMP9 [48], and Wang et al. demonstrated that EPS11 reduced tumor nodule formation in lungs in vivo, supporting the anti-metastatic potential of bacterial polysaccharides [49]. This difference indicates that the substance selectively inhibits cancer cell migration and promotes normal cell healing.

## 3. Conclusions

EPS was extracted from *Bacillus amyloliquefaciens* SQ-2 by ethanol precipitation, TCA deproteinization, and column chromatography. The purified fraction EPS-3791 was identified as a fructan by methylation analysis and NMR spectroscopy. The predominant glycosidic linkage was →1)–Fruf–(2→ (molar ratio 0.777), with additional linkages including Fruf–(2→, Glcp–(1→, →6)–Glcp–(1→, and →3,6)–Glcp–(1→. The molecular weight was 64.41 kDa with a PDI value of 1.6, indicating moderate molecular heterogeneity. Further fractionation will be performed in future studies to obtain a more homogeneous polysaccharide preparation. SEM showed a porous three-dimensional network, and XPS indicated an elemental composition of C, O, and trace N. EPS-3791 aerogels were fabricated by freeze drying the purified EPS, directly yielding the porous scaffold form. In vitro bioassays demonstrated that the aerogels exhibited no cytotoxicity toward normal L929 cells, while dose-dependently inhibiting the proliferation of A549, MCF–7, and HeLa cancer cells, with A549 cells showing the highest sensitivity. The scratch wound healing assay showed that EPS-3791 aerogel inhibited HeLa migration and promoted L929 wound closure. These results identify EPS-3791 as a fructan-type exopolysaccharide with selective anticancer activity. Determination of the underlying molecular mechanisms and in vivo validation are needed to assess its therapeutic potential.

## 4. Materials and Methods

### 4.1. Strains and Bacterial Growth

The biocontrol potential of *Bacillus amyloliquefaciens* SQ-2, a probiotic strain isolated from commercial broad bean paste, against *Aspergillus tubingensis*-caused table grape rot has previously been investigated [9]. The strain was maintained in LB liquid medium supplemented with 20% (*v*/*v*) glycerol at −80 °C. The strain SQ-2 was cultured at a pH of 8.0, a temperature of 25 °C, and a speed of 200 r/min for 24 h. Subsequently, extraction and purification of EPS were performed.

### 4.2. Extraction and Purification of EPS

Crude EPS was extracted from the fermentation broth of strain SQ-2 according to a previously reported method [50]. Cells were removed by centrifugation at 8000× *g* for 15 min. Trichloroacetic acid (TCA) was added to the supernatant to a final concentration of 10% (*v*/*v*), and the precipitated proteins were removed by centrifugation at 10,000× *g* for 10 min. Four volumes of cold ethanol (80%, *v*/*v*) were then added to the supernatant, and the mixture was kept at 4 °C overnight. To obtain crude EPS, named EPS-1, the precipitate was collected by centrifugation at 10,000× *g* for 10 min, dissolved in ultrapure water, and dialyzed against ultrapure water for 3 days using a 14,000 Da molecular weight cutoff dialysis membrane. The dialysate was renewed at 0.5 h, 1 h, and 4 h initially. Then it was renewed every 12 h. Finally, it was followed by freeze drying. The fermentation yield of EPS was calculated according to the following formula:

(1) $$Y_{EPS}=m/V$$

where $Y_{EPS}$ represents the EPS yield (mg/L), m is the mass of lyophilized EPS (mg), and V is the volume of fermentation broth (L).

The initial purification of EPS-1 was carried out using anion exchange chromatography. After passing through a (20 mm × 200 mm) DEAE–Sepharose fast flow column, the EPS-1 was eluted at a rate of 2.0 mL/min using a gradient of 0–9% NaCl solution. The phenol–sulfuric acid colorimetric method was used to determine the amount of carbohydrates of each fraction (~5 mL) [51]. Using ultrapure water as the eluent at a flow rate of 0.3 mL/min, gel filtration chromatography was used for the additional purification on a Sephadex G-100 column (10 mm × 500 mm). Approximately 5 mL fractions were collected. The primary fractions were combined, dialyzed, and freeze dried to yield pure EPS (named EPS-3791) for further analysis.

### 4.3. Structural Characterization of EPS-3791

#### 4.3.1. Purity Identification

The purified EPS was dissolved in ultrapure water to form a 1 mg/mL solution. EPS-3791’s total carbohydrate content was determined using the phenol–sulfuric acid colorimetric method, with glucose serving as the reference standard.

#### 4.3.2. Monosaccharide Composition Determination

Ion chromatography was used to analyze the monosaccharide content of EPS-3791. The 5 mg of EPS-3791 and standard monosaccharides were hydrolyzed with trifluoroacetic acid (TFA). The reaction mixture was dried under a stream of nitrogen and washed three times with methanol. To facilitate detection, the residue was dissolved in sterile water. The release fragments were detected on a Thermo ICS–5000 ion chromatograph system (Thermo Fisher Scientific, Waltham, MA, USA) coupled with a Dionex^TM^ CarboPac^TM^ PA20 liquid chromatographic column (150 mm × 1.0 mm, 10 μm. Thermo Fisher Scientific, Waltham, MA, USA) and a Dionex ED50A electrochemical detector (Thermo Fisher Scientific, Waltham, MA, USA) [52]. The temperature was 30 °C and the flow rate was 8 μL/min. For identification and quantification, sixteen monosaccharides were used as reference standards: fucose, arabinose, rhamnose, galactose, glucose, xylose, mannose, galacturonic acid, gluconic acid, D-ribose, D-fructose, L-guluronic acid, N-acetyl–D-glucosamine, glucosamine hydrochloride, galactosamine hydrochloride, and D-mannuronic acid.

#### 4.3.3. Fourier Transform Infrared (FTIR) Spectroscopy Analysis

An IRTracer–100 Fourier transform infrared spectrometer (FT–IR, IRTracer–100, Shimadzu Corporation, Kyoto, Japan) was used to conduct functional group analysis. The purified EPS was mixed with dried potassium bromide (KBr) powder at a mass ratio of 1:100 (sample: KBr, *w*/*w*), ground thoroughly, and pressed into a transparent pellet. The wavenumber range of 4000–400 cm^−1^ was used to acquire FTIR spectra.

#### 4.3.4. Methylation Analysis

Permethylation derivatization was used to study the glycosidic linkage of EPS-3791, and gas chromatography–mass spectrometry (GC–MS) was used to examine the partially methylated alditol acetates (PMAAs) that were produced [53]. 

#### 4.3.5. Nuclear Magnetic Resonance (NMR) Analysis

Lyophilized EPS-3791 (30 mg) was dissolved in 0.5 mL of D_2_O and lyophilized; this process was repeated three times to facilitate the exchange of labile protons with deuterium. After being redissolved in D_2_O, the sample was moved to an NMR tube and analyzed at 298 K using a Bruker 800 MHz spectrometer (Avance III–800 MHz, Bruker, Fällanden, Switzerland) [54]. Data were processed using MestReNova (v15.0) software.

#### 4.3.6. Microstructural Analysis of SQ-2 and EPS-3791

Scanning electron microscopy was used to investigate lyophilized EPS-3791 and dried bacterial cells after they had been thinly coated with gold (SEM, FEI–Verios 460 L, Thermo Fisher Scientific, Waltham, MA, USA). Surface micrographs of both the cells and the EPS were acquired. Accelerating voltages were 10 kV for the polysaccharide samples and 3 kV for the bacterial cells [55]. EPS-3791 was dissolved in absolute ethanol to prepare a 60 μg/mL solution and sonicated for 45 min. Approximately 5 μL of the polysaccharide solution was dropped onto the center of a mica sheet and dried in a dust-free environment for at least 2 h, until the surface appeared completely dry. Surface morphology of EPS-3791 was obtained by atomic force microscopy (AFM, Dimension icon, Bruker, Karlsruhe, Germany).

#### 4.3.7. XPS Analysis

Elements and chemical bond properties of EPS-3791 were ascertained by means of X-ray photoelectron spectroscopy.

#### 4.3.8. TG Analysis

The thermal stability of EPS-3791 between 40 and 800 °C was evaluated using the thermogravimetric analyzer (TGA).

### 4.4. Preparation of EPS-3791 Aerogels by Freeze Drying

EPS-3791 aerogels were prepared by freeze drying according to a previously described method [15]. Approximately 2 mL of a 1% (*w*/*v*) aqueous EPS-3791 solution was pipetted into glass vials and frozen at −80 °C. To create porous aerogel monoliths, the frozen samples were subsequently moved to a freeze-dryer (LGJ-10, Sihuan Furuikeyi Technology Development Co., Ltd., Beijing, China) and lyophilized for 48 h at −50 °C and 0.31 mbar. The yield of the aerogel was calculated using the following equation:

(2) $$Y_{Ag}=m/M\times 100\&\%$$

where $Y_{Ag}$ is the yield of the aerogel (%), m is the mass of the final aerogel product, and M is the initial mass of the raw material.

### 4.5. Impact of EPS-3791 Aerogel on Cell Proliferation

HeLa, A549, and MCF–7 are common typical tumor cell models for the activity screening of microbial EPS, and L929 mouse fibroblast cells were applied as normal control cells to verify its biosafety [56,57]. This study mainly focuses on the selective inhibitory effects and biosafety of EPS-3791 aerogel against various tumor cells. Based on the above considerations, four cell lines were selected to evaluate the antitumor activity of EPS-3791 aerogel. HeLa, MCF–7, A549, and L929 cells were plated at 100 μL per well in 96-well plates after being diluted to 1.0 × 10^4^ cells/mL. EPS-3791 aerogel distributed in serum-free culture medium at doses of 50, 100, 200, 400, and 800 μg/mL was used in place of the media after a 24 h incubation period; medium-only wells were used as the blank control. After 24 h or 48 h of treatment, 20 μL of MTT solution was added per well. Plates were shaken for 10 min, then incubated for 4 h. Cell viability was calculated as:

(3) $$Cellviability\left(\%\right)=\frac{A_{test}}{A_{control}}\times 100\%$$

where $A_{control}$ is the absorbance value of untreated cells at 490 nm and $A_{test}$ is the absorbance value of treated cells at 490 nm.

### 4.6. Cell Scratch Assay

Cell migration was assessed in vitro by the scratch (wound healing) assay using L929, A549, MCF–7, and Hela cells [58]. Uniform parallel reference lines were marked on the back of 6-well plates. After serum starvation for 6 h, scratches were made along the reference lines using a sterile pipette tip. After scratching, cells were washed three times with sterile PBS to remove detached cells, ensuring a clear wound gap. Fresh serum-free medium was added to each well. The experimental group received EPS-3791 aerogel dissolved in serum-free medium at 10 mg/mL, and plates were incubated at 37 °C in a CO_2_ incubator. Images were captured at 24, 48 and 72 h using an inverted microscope at 100× magnification. Migration rate was evaluated based on the wound width in scratch micrographs after treatment.

### 4.7. Statistical Analysis

The data is expressed as the mean ± standard deviation (SD) of three replicates. To ascertain whether there were significant differences in the parameter means, one-way ANOVA and Duncan’s multiple-range test were employed using SPSS software (v26.0, SPSS, Inc. Chicago, IL, USA). *p* < 0.05 was established as the cutoff point for statistical significance.

## Data Availability

The original contributions presented in this study are included in the article. Further inquiries can be directed to the corresponding author.

## Supplementary Materials

The following supporting information can be downloaded at https://www.mdpi.com/article/10.3390/gels12060462/s1, Figure S1. FTIR spectrum of EPS–3791 in the wavenumber range of 4000–500 cm^–1^. Figure S2. GC–MS of EPS–3791. Figure S3. Types of glycosidic bonds in EPS–3791. Figure S4. Proposed schematic structure of the EPS–3791 fructan chain. Figure S5. Particle size distribution of EPS–3791 aggregates. Figure S6. Pore size distribution of EPS–3791 aggregates. Figure S7. TG and DTG curves of EPS–3791. Table S1. Monosaccharide composition of EPS-3791 analyzed by HPAEC-PAD. Table S2. Methylation analysis of EPS–3791 polysaccharide. Table S3. Partially Methylated Alditol Acetates (PMAA) Analysis of EPS–3791 Polysaccharide.

## Abbreviations

The following abbreviations are used in this manuscript:

EPS	Exopolysaccharide
HPAEC–PAD	High-performance anion exchange chromatography with pulsed amperometric detection
NMR	Nuclear magnetic resonance
FTIR	Fourier transform infrared spectroscopy
SEM	Scanning electron microscopy
AFM	Atomic force –microscope
XPS	X-ray photoelectron spectroscopy
TGA	Thermogravimetric analysis
MTT	3–(4,5–dimethylthiazol–2–yl)–2,5–diphenyltetrazolium bromide
TFA	Trifluoroacetic acid
