# Supplementary material for: Fabrication and Characterization of Bio-Based Aerogels Derived from Bacillus amyloliquefaciens SQ-2 Exopolysaccharides: Structural Characterization and In Vitro Antitumor Activity Analysis
Source: Gels. 2026 May 25;12(6):462. doi: 10.3390/gels12060462 (PMC13297979; doi:10.3390/gels12060462)
Supplement: Supplementary file 1 [file gels-12-00462-s001.zip › gels-4245484-supplementary.pdf]

[Supplementary information]

Fabrication and Characterization of Bio-Based Aerogels Derived from *Bacillus amyloliquefaciens* SQ-2 Exopolysaccharides: Structural Characterization and In Vitro Antitumor Activity Analysis

Tianjiao Zhao [1,†], Lei Huang [1,†], Sihan Wei [1], Chengci Liu [1], Jinhua Xu [2], Lu Qiao [2,3], Jincheng Li [2], Chaoying Zhang [2], Yingchun Mu [2], Zhiyang Zhao [4], Meitong Li [1,*] and Xin Hu [2,*]

[1] Tianjin Key Laboratory of Organic Solar Cells and Photochemical Conversion, College of Chemistry and Chemical Engineering, Tianjin University of Technology, Tianjin 300384, China; tianjiaozhao@stud.tjut.edu.cn (T.Z.); huanglei@tjut.edu.cn (L.H.); yj20251014@stud.tjut.edu.cn (S.W.); chengciliu@163.com (C.L.)

[2] Chinese Academy of Fishery Sciences, Beijing 100141, China; xujh@cafs.ac.cn (J.X.); qiaolu@cafs.ac.cn (L.Q.); lijc@cafs.ac.cn (J.L.); cyzhang0317@cafs.ac.cn (C.Z.); muyc@cafs.ac.cn (Y.M.)

[3] Hainan Fisheries Innovation Research Institute, Chinese Academy of Fishery Sciences, Sanya 572000, China

[4] Jiangsu Key Laboratory of New Energy Devices & Interface Science, School of Chemistry and Materials Science, Nanjing University of Information Science and Technology, Nanjing 210044, China; zhzh@nuist.edu.cn

* Correspondence: tjutlmt@email.tjut.edu.cn (M.L.); huxin@cafs.ac.cn (X.H.)

† These authors contributed equally to this work.

10 Pages including cover page

7 Figures

3 Table

List of Figures and Tables

Figure S1. FTIR spectrum of EPS–3791 in the wavenumber range of 4000–500 cm^{-1}.

Figure S2. GC–MS of EPS–3791.

Figure S3. Types of glycosidic bonds in EPS–3791.

Figure S4. Proposed schematic structure of the EPS–3791 fructan chain.

Figure S5. Particle size distribution of EPS–3791 aggregates.

Figure S6. Pore size distribution of EPS–3791 aggregates.

Figure S7. TG and DTG curves of EPS–3791.

Table S1. Monosaccharide composition of EPS-3791 analyzed by HPAEC-PAD.

Table S2. Methylation analysis of EPS–3791 polysaccharide.

Table S3. Partially Methylated Alditol Acetates (PMAA) Analysis of EPS–3791 Polysaccharide

Figure S1. FTIR spectrum of EPS–3791 in the wavenumber range of 4000–500 cm^{-1}.

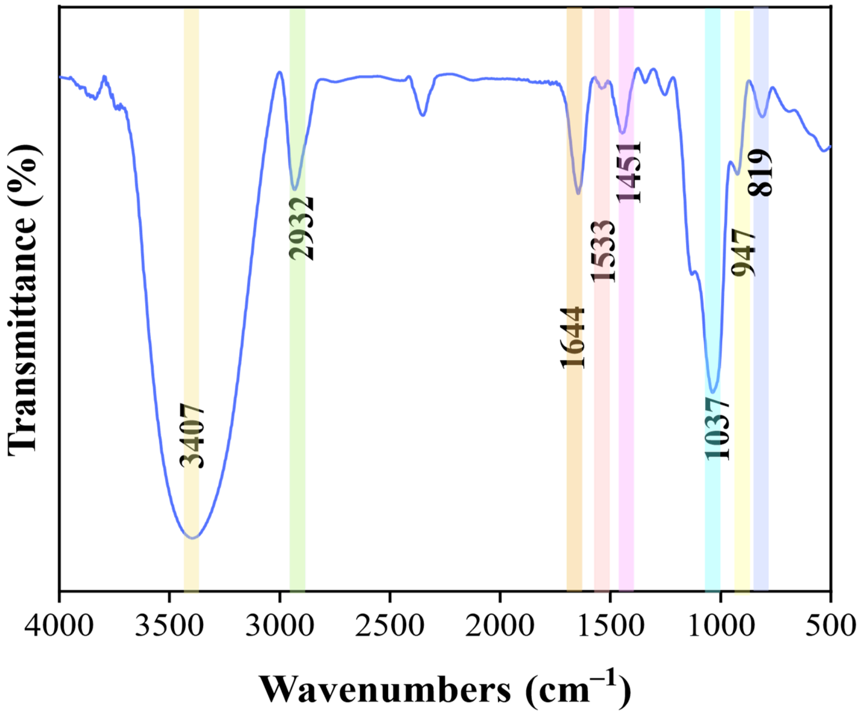

Figure S2. GC–MS of EPS–3791.

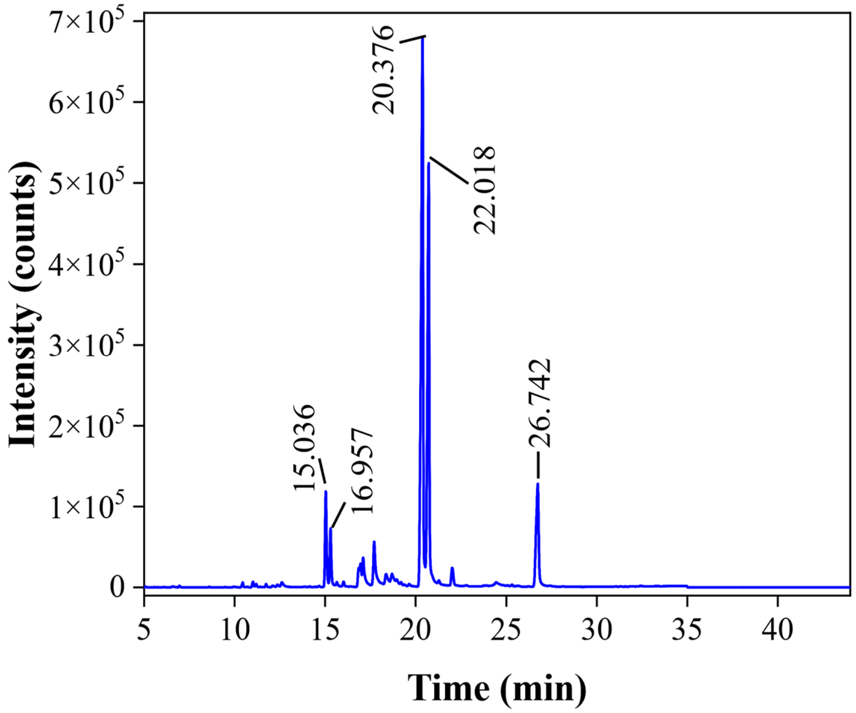

Figure S3. Types of glycosidic bonds in EPS–3791.

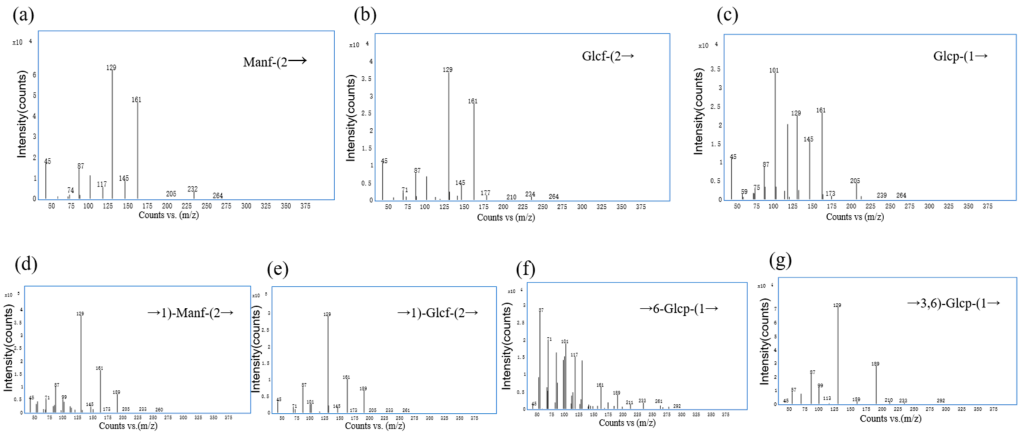

Figure S4. Proposed schematic structure of the EPS–3791 fructan chain.

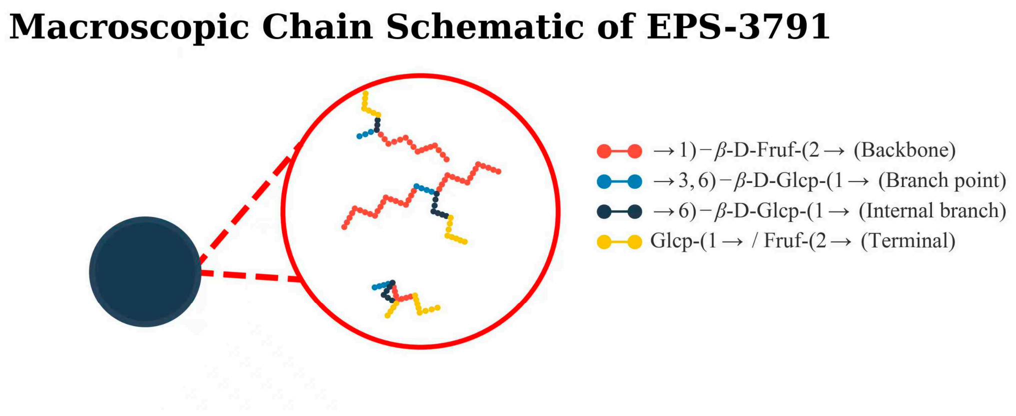

Figure S5. Particle size distribution of EPS–3791 aggregates.

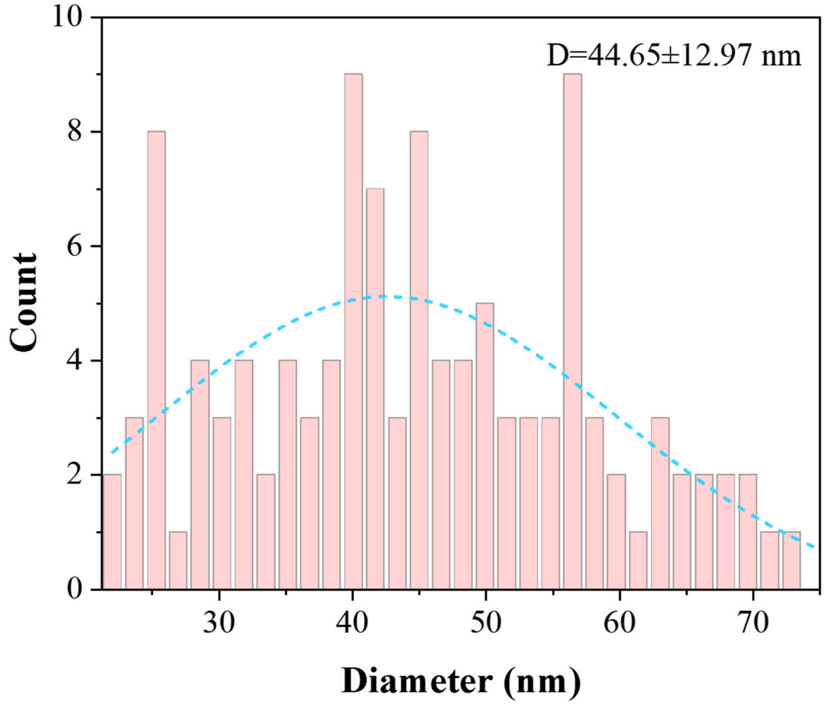

Figure S6. Pore size distribution of EPS–3791 aggregates.

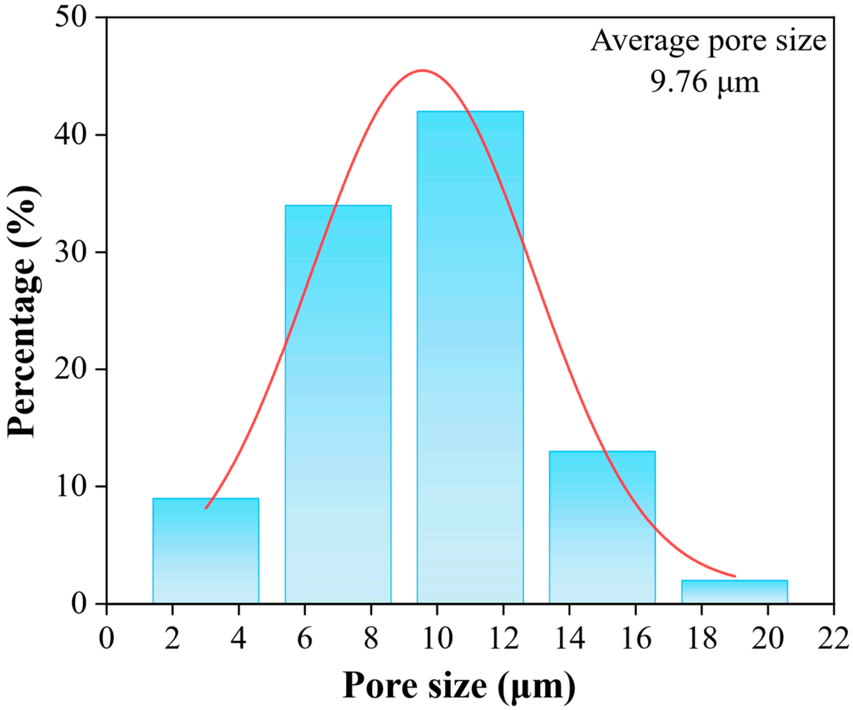

Figure S7. TG and DTG curves of EPS–3791.

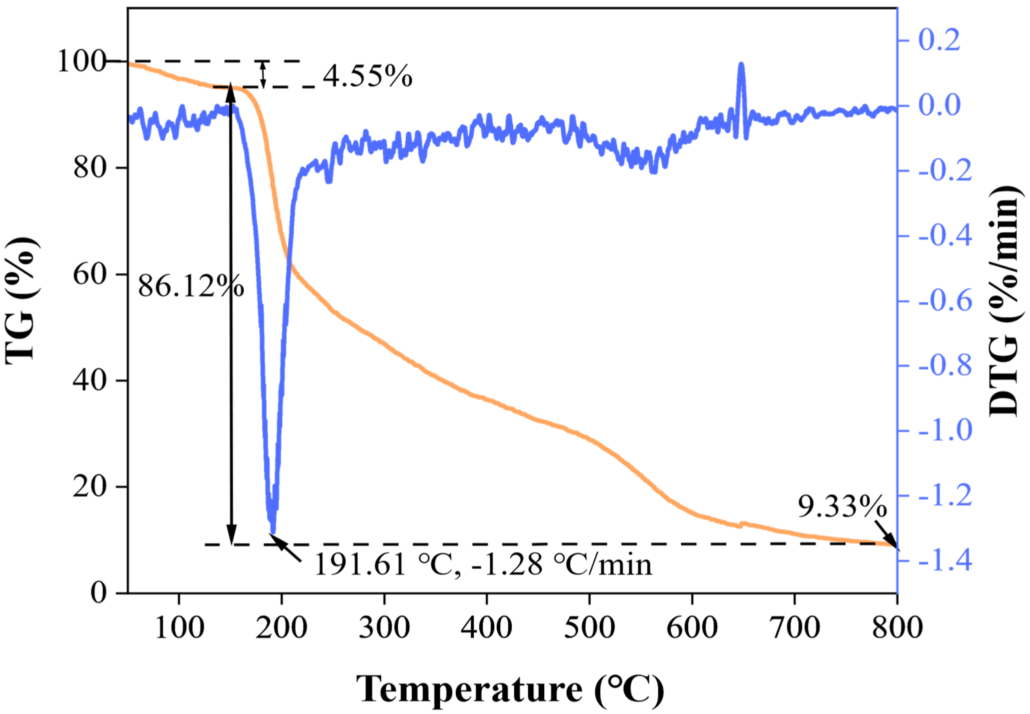

Table S1. Monosaccharide composition of EPS-3791 analyzed by HPAEC-PAD.

Name	Peak area	Retention time	Molar ratio (%)
Fucose	0	6.117	0.000
Galactosamine hydrochloride	0	11.209	0.000
Rhamnose	0	11.734	0.000
Arabinose	0	12.225	0.000
Glucosamine hydrochloride	0	13.925	0.000
Galactose	0	15.209	0.000
Glucose	0.651	17.35	0.007
N–Acetyl–D–glucosamine	0	19.275	0.000
Xylose	0	20.009	0.000
Mannose	0	20.725	0.000
Fructose	13.148	23.150	0.993
Ribose	0	25.509	0.000
Galacturonic acid	0	44.942	0.000
Guluronic acid	0	45.617	0.000
Glucuronic acid	0	48.000	0.000
Mannuronic acid	0	50.742	0.000

Table S2. Methylation analysis of EPS–3791 polysaccharide.

RT	Methylated sugar	Mass fragments (m/z)	Molar ratio	Type of linkage
15.036	1,3,4,5–Me_4–Manf/Glcf	87,101,129,145,161	0.095	Fruf–(2→

16.957	2,3,4,6–Me4–Glcp	43,71,87,101,117,129,145,161,205	0.015	Glcp–(1→
20.376	3,4,5–Me3–Manf/Glcf	43,71,87,99,101,129,145,161,189	0.777	→1)–Fruf–(2→
22.018	2,3,4–Me3–Glcp	43,87,99,101,117,129,161,189,233	0.014	→6)–Glcp–(1→
26.742	2,4–Me2–Glcp	43,87,117,129,159,189,233	0.099	→3,6)–Glcp–(1→

Table S3. Partially methylated alditol acetates (PMAA) analysis of EPS–3791.

RT	Methylated sugar	Mass fragments (m/z)	Molar ratio	Type of linkage
15.036	1,3,4,6–Me_4–Manf	87,101,129,145,161	0.063	Manf–(2→
15.298	1,3,4,6–Me_4–Glcf	87,101,129,145,161	0.039	Glcf–(2→
16.957	2,3,4,6–Me_4–Glcp	43,71,87,101,117,129,145,161,205	0.016	Glcp–(1→
20.376	3,4,6–Me_3–Manf	43,71,87,99,101,129,145,161,189	0.455	→1)–Manf–(2→
20.714	3,4,6–Me_3–Glcf	43,71,87,99,101,129,145,161,189	0.311	→1)–Glcf–(2→
22.018	2,3,4–Me_3–Glcp	43,87,99,101,117,129,161,189,233	0.015	→6)–Glcp–(1→
26.742	2,4–Me_2–Glcp	43,87,117,129,159,189,233	0.100	→3,6)–Glcp–(1→
